# Profiles of family caregivers of patients at the end of life at home: a Q-methodological study into family caregiver’ support needs

**DOI:** 10.1186/s12904-020-00560-x

**Published:** 2020-04-21

**Authors:** Femmy M. Bijnsdorp, H. Roeline W. Pasman, Cécile R. L. Boot, Susanne M. van Hooft, AnneLoes van Staa, Anneke L. Francke

**Affiliations:** 1 Amsterdam UMC, Vrije Universiteit Amsterdam, Department of Public and Occupational Health, Amsterdam Public Health research institute, Expertise Center for Palliative Care, P.O. Box 7057, 1007 MB Amsterdam, The Netherlands; 2 Amsterdam UMC, Vrije Universiteit Amsterdam, Department of Public and Occupational Health, Amsterdam Public Health research institute, Amsterdam, The Netherlands; 3 Research Centre Innovations in Care, Rotterdam University, Rotterdam, The Netherlands; 4 Erasmus School of Health Policy & Management, Erasmus University Rotterdam, Rotterdam, The Netherlands; 5 Nivel, Netherlands institute for health services research, Utrecht, The Netherlands

**Keywords:** Caregiver’ profiles, End of life, Family caregivers, Q-methodology, Support needs

## Abstract

**Background:**

Family caregivers of patients at the end of life often experience care-related burden. To prevent caregiver burden and to enhance the capacity to provide care it is important to have insight in their support needs. The purpose of this study was to identify profiles of family caregivers who provide care to patients at the end of life at home.

**Methods:**

A Q-methodological study was conducted in which family caregivers ranked 40 statements on support needs and experiences with caregiving. Thereafter they explained their ranking in an interview. By-person factor analysis was used to analyse the rankings and qualitative data was used to support the choice of profiles. A set of 41 family caregivers with a variety on background characteristics who currently or recently provided care for someone at the end of life at home were included.

**Results:**

Four distinct profiles were identified; profile (1) those who want appreciation and an assigned contact person; profile (2) was bipolar. The positive pole (2+) comprised those who have supportive relationships and the negative pole (2-) those who wish for supportive relationships; profile (3) those who want information and practical support, and profile (4) those who need time off. The profiles reflect different support needs and experiences with caregiving.

**Conclusions:**

Family caregivers of patients at the end of life have varying support needs and one size does not fit all. The profiles are relevant for healthcare professionals and volunteers in palliative care as they provide an overview of the main support needs among family caregivers of patients near the end of life. This knowledge could help healthcare professionals giving support.

## Background

Taking care of someone near the end of life is often difficult; one in ten family caregivers who provide care to a patient at the end of life experiences care-related burden [1]. Burden can be particularly high for family caregivers of patients approaching the end of life in the home setting, as the patient becomes more dependent on them. In addition, family caregivers could experience feelings of grief due to the impending death of their relative [1, 2]. The likelihood of experiencing heavy burden was found to increase from 32% in the second and third months before death to 66% 1 week before death among family caregivers ‘at home’ [3]. Burden can result in physical and psychological morbidity [4, 5], restrictions on the family caregiver’s own life and a strain on financial resources [6, 7]. Older family caregivers, those caring for a partner and those caring for a person with dementia are particularly at risk of physical and psychological health issues during end-of-life care [8, 9]. It is important that family caregivers obtain support in good time to prevent them from becoming overloaded and to enhance their capacity to provide care. This could also be relevant for the quality of life, and ultimately, the quality of the patient’s final days.

Healthcare professionals such as nurses, home-care staff and volunteers have various ways in which they can support family caregivers. In general, these can be divided into two domains:

(1) support to enable family caregivers to provide care (the caregiver as ‘co-worker’) [10], such as practical help with care tasks, coordination of care, providing information [11–14] and support in managing symptoms or giving medicines [11, 15, 16]; and (2) psychosocial support aimed at improving the wellbeing of the family caregiver (the caregiver as ‘co-client’) [10], such as respite care (e.g. day-care programmes to give family caregivers temporary relief) [17], emotional support and attending to social needs [11–14]. A recent study among family caregivers of people with dementia has shown that the top five support needs were more time off, knowing what to expect in the future, practical help in the home, support with their own health, and dealing with feelings and worries. Most of these support needs are related to direct support for the family caregiver rather than support to help them provide care, implying that family caregivers should also be seen as co-clients [18]. However, healthcare professionals generally have a more client-centred approach in which they focus on the needs of the patient, and the family caregiver’s support needs can easily be overlooked [18, 19]. Thus, despite efforts made by healthcare professionals and volunteers, family caregivers’ support needs often remain unmet [8, 14, 20]. Previous studies have shown that unmet support needs included social needs (e.g. financial support), cognitive needs (e.g. support in decision-making and managing concerns about the disease) and psychological needs (e.g. support in mourning and addressing fears) [14, 20].

However, the specific (unmet) support needs may vary greatly between family caregivers and over time. To date, it remains unclear what kinds of viewpoints exist regarding preferred support and experiences with the situation in terms of burden, positive experiences, obstacles and received support among people who provide care to patients at the end of life. Former studies of family caregivers’ support at the end of life have generally been limited to specific disease groups [5, 13–15, 18, 21–23] or support options [17, 24]. Also, support needs and experiences with the care situation are often assessed using a cross-sectional approach, which mostly results in a number of support preferences and experiences from a majority perspective. However, majority viewpoints do not reflect the variation in support needs and experiences among family caregivers. Hence, differences between family caregivers in their specific support needs and experiences with caregiving remain neglected. The aim of this study is to identify profiles of family caregivers who provide care to patients at the end of life in the home setting, as distinguished by their support needs and experiences with caregiving. These profiles could help healthcare professionals and volunteers to recognize various target groups and could shed light on how they can support them. Also, the profiles could offer a starting point to discuss specific caregivers’ needs. Timely and appropriate support could help family caregivers and improve end-of-life care for patients.

## Methods

### Q-methodology

**Fig. 1:**
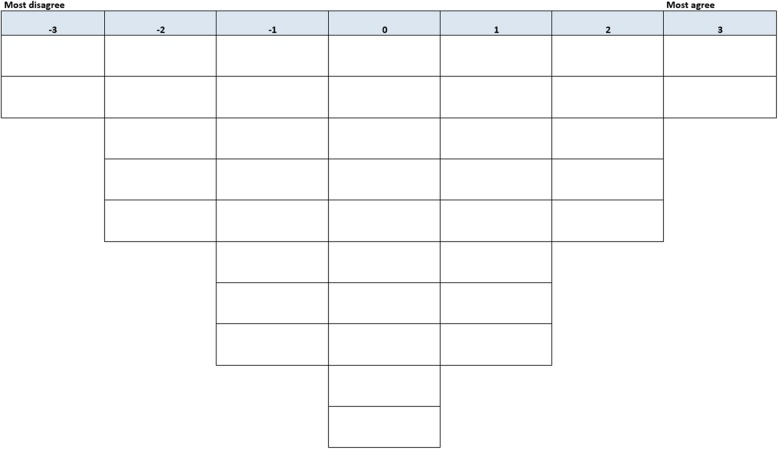
Forced choice frequency distribution in Q-sort**.** Participants ranked the cards with the statements on this score sheet. The cards with the two statements they agreed most with were placed in the rightmost part of the score sheet below ‘most agree’, working their way through until all the statements in this category were ranked. They repeated the process with the statements they disagreed with (starting in the leftmost part of the distribution) and the neutral statements (placing the statements in the empty spaces in the distribution)

We used Q-methodology to identify family caregiver profiles. In recent years, this method has been widely used in the field of healthcare research [17, 22, 25–31]. Q-methodology combines qualitative and quantitative research methods in order to identify groups of persons who share similar viewpoints about a specific topic and therefore belong to a certain type or profile [32, 33]. To determine these profiles, participants are presented with a heterogeneous set of statements about the topic. This group of statements is called a Q-sample [34]. The statements must be ranked relative to all other statements in the distribution provided (see Fig. 1). This is known as the Q-sort, and is often a quasi-normal distribution [32]. The individual rankings are then subjected to a by-person factor analysis. The correlation between participants indicate similarities or differences in viewpoints on a specific topic [34, 35].

### Formulating the statements (Q-sample)

In order to capture relevant aspects of family caregivers’ support needs and experiences with caregiving, we collected statements about this subject using existing scales and questionnaires, scientific literature and reports. The Carer Support Needs Assessment Tool (CSNAT) was an important component in the design of the Q-sample in that we divided all support-related items into the categories ‘support to enable the family caregiver to provide care’ or ‘support for the family caregivers themselves’. [10] The CSNAT is an evidence-based tool for the assessment of family caregivers’ support needs in multiple domains and was developed for use in palliative care [10, 19]. In addition, statements from earlier Q-methodological studies of the attitudes and beliefs of dementia caregivers [22] and respite care [17] were examined, as well as other studies of caregivers’ support needs [2, 15], self-perceived burden [36] and positive experiences with caregiving [37]. This resulted in a list of 118 statements. To obtain a representative sample of statements, we first classified them by theme. We then chose a subset of 40 statements by eliminating duplicates and similar statements (see Table 1). After that, we pre-tested the Q-sample with three former family caregivers to check that the statements were clear and understandable and to check whether there were other important viewpoints or topics that we had not included. Based on their input, two statements were rephrased to make them more understandable (item 14 and 17). After that, we randomly assigned a number to each statement.

**Table 1:** Statements by themes represented in final Q-sample

Major themes	Sub-themes	Statements
Support domains to enable the family caregiver to provide care (co-worker items)		
I.	Support for care	13, 22, 26, 28
II.	Coordination of care	14, 35, 36, 27
III.	Need for information	24, 29, 38
Support domains for family caregivers themselves (co-client items)		
IV.	Emotional support	16, 32, 39
V.	Respite care	3, 12, 30
VI.	Social needs	4, 11, 25, 33
VII.	Capacity as caregiver	5, 34, 40
Experiences with care situation		
VIII.	Experienced pressure (light-moderate)	9, 19, 21, 31
IX.	Experienced burden (severe)	6, 18, 23
X.	Experienced support	8, 15, 17, 20, 37
XI.	Experienced obstacles	2, 10
XII.	Positive experiences	1, 7

### Participants and data collection

In this paper, ‘family caregiver’ refers to unpaid persons providing physical, practical and/or emotional care and support to a relative or friend [38]. Family caregivers were recruited via the newsletters, websites and social media channels of Dutch national organizations of family caregivers and volunteers in palliative care. We selected all family caregivers who had recently (< 2 years) provided care for patients at the end of life in the home-setting (e.g. patients who had been diagnosed with incurable cancer, chronic obstructive pulmonary disease (COPD), heart failure, dementia, stroke, neurodegenerative disorder or severe general decline). Forty-three family caregivers signed up for participation. However, two did not meet the selection criteria: for one of them the care was five years ago and one provided care to someone in a nursing home. Hence, 41 current and recent family caregivers were included. They varied in their background characteristics (e.g. age, relationship and illness type). In contrast to traditional quantitative research, Q-methodology does not require large numbers of participants, because the aim is to identify shared viewpoints and not to provide information on the distribution of these viewpoints in the population [32, 39]. A number of participants between 40 and 60 is considered to be more than sufficient [39], while Q-studies can be carried out with far fewer [32, 40].

The interviews were held in the homes of the family caregivers. The participants were asked to say something about the care situation. After that, the statements of the Q-sample were presented. The statements were printed on separate cards for ranking on the Q-sort. Participants were asked to sort the statements into three categories: statements they definitely agreed with, statements they definitely disagreed with and neutral statements or statements that did not apply to them [32]. Next, they sorted the statements they definitely agreed with and allocated them on the Q-sort distribution, starting with the two statements they agreed with most in the rightmost part of the distribution and working their way through until all the statements in this category were ranked. They repeated the process with the statements they disagreed with (starting in the leftmost part of the distribution) and the neutral statements (placing the statements in the empty spaces in the distribution). After the sorting, the participants were asked if they were satisfied with the configuration. When satisfied, participants were interviewed face-to-face to explain their choice for the statements (see the interview guide in Additional file 1). All comments during the sorting process and the post-sorting interviews were audio-recorded and used for the interpretation of the factor arrays and creation of the profiles. The use of post-sorting interviews can improve the validity as they reveal participants’ reasoning and this increases the richness and quality of the data [32, 41].

### Caregiver context

After agreeing to participate, family caregivers filled in a short survey assessing individual characteristics (e.g. sex, age, education, country of birth and their parents’ country of birth) and their care situation. The participants provided information about their relationship to the patient (e.g. partner, parent, son/daughter, brother/sister, other relative, neighbour/friend), the diagnosis (e.g. incurable cancer, COPD, heart failure, dementia, stroke, neurodegenerative disorder or other) and contact frequency (lives in same household, daily contact, weekly or monthly). In addition, we asked whether they received support with the care and from whom (e.g. from their social network, home-care staff or volunteers). Because caregiving is often not the only societal role family caregivers have [42], we asked whether they had a paid job when the family care started (yes/no). If they answered ‘yes’, we asked how many hours they worked per week (on average) when the family care started and whether they had adjusted their work situation because of the family care.

### Data analysis

All the individual rankings were intercorrelated and subjected to a by-person factor analysis (centroid factor extraction with varimax rotation) using the dedicated computer package PQMethod [43]. The objective of the analysis was to identify similarities in how the family caregivers ranked the statements. Family caregivers with the same views shared the same factor. The final factor solution was selected by calculating factor loadings to determine which Q-sorts load onto each factor and by investigating the qualitative data to assess support for the factor solution [29]. Factors were retained if they had an eigenvalue > 1, had at least two Q-sorts that loaded onto the factor and were coherent, differentiated, interpretable and meaningful in a qualitative manner [32, 44]. A theoretical Q-sort was computed for each factor; this is the Q-sort that represents the viewpoint and is based upon the Q-sorts of those identified on this view (factor). The factors were interpreted as family caregiver profiles and described using the theoretical Q-sorts. A factor array of + 3 means that the participant most agreed with this statement and a score of − 3 that he or she most disagreed with that statement (also see Fig. 1). A first impression of the profiles was based on the characterizing statements (those with a factor array of + 3, + 2, − 2 or − 3), the distinguishing statements (those with a factor array that was statistically significantly different to the scores in all other factors) and the consensus statements (those that do not distinguish between any pair of factors). The qualitative data from the caregivers who significantly loaded onto the factors was analysed to understand why they most agreed or disagreed with these statements. In this way, the qualitative material added meaning to the factors. The compiled rankings together with the qualitative material from the family caregivers loading on a factor were used for further interpretation of the profiles.

## Results

The background characteristics of the study participants are presented in Table 2. We found four meaningful distinctive profiles of family caregivers who provide care to patients at the end of life at home (see Table 3). The four profiles were defined by between four and nine Q-sorts each (27 in total) and explained between 9 and 11% of the variance in the Q-sorts, 40% in total. Twelve Q-sorts did not load on any factor and two Q-sorts loaded on two factors. In the following descriptions of the profiles, the statement numbers are given between brackets and the quotes from the family caregivers are noted in italics. The main characteristics of each profile are presented in Table 4.

**Table 2:** Characteristics of study participants (*n* = 41)

	n (%)
Sex (female)	35 (85.4)
Age [range 26–81] (M, SD)	61.1 (±12.5)
Educational level (primary, secondary, tertiary)	5 (12.2)/ 12 (29.3)/ 24 (58.5)
Cultural background	
Dutch (native)	31 (75.6)
Non-Dutch (Western)	6 (14.6)
Non-Dutch (non-Western)	4 (9.8)
Has provided family care in the past	26 (63.7)
Currently provides family care	15 (36.3)
Provides/provided care for	
Partner	20 (48.8)
Parent	16 (39)
Other	5 (12.2)
Type of illness	
Cancer	16 (39)
Dementia	16 (39)
Organ failure	13 (31.7)
Stroke (CVA)	5 (12.2)
Other ^a^	8 (19.5)
Contact frequency with care recipient	
Lives in same house	23 (56.1)
Daily contact	16 (39)
Weekly contact	2 (4.9)
Received support from	
Own network	28 (68.3)
Home-care staff	30 (73.2)
Volunteers	6 (14.6)
Other ^b^	5 (12.2)
No support	2 (4.9)
Paid work when family care started (yes)	23 (56.1)
Work hours per week [range 10–66] (M, SD)	32.2 (±12.7)
Adjusted work situation because of family care (yes) ^c^	14 (60.9)
Quit job	10 (43.5)
Work adjustments ^d^	7 (30.4)
Care/sick leave	4 (17.4)

Note: ^a^ Other diseases or problems that were mentioned included progressive neurologic disorder, prolapse, dehydration, urinary infection, delirium, open wounds, multiple falling accidents and posttraumatic stress disorder (war trauma). ^b^ Other support included dementia support groups, case managers and privately paid caregivers. ^c^ Percentage of working family caregivers. ^d^ Work adjustments included reduced working hours, flexible working hours and working from home

**Table 3:** Statements, factor arrays, explained variance and eigenvalues

Statements		Profiles			
		1	2	3	4
1	Looking after my relative gives me a good feeling	3	2	2*	−3**
2	It is difficult to set your own boundaries and stand up for yourself	3	1	1	2
3	I need more support with practical help in the home	0	−1	0	−1
4	I need more support with my financial, legal or work issues	−1	0**	−2	−2
5	I appreciate it when someone asks me how I am myself	2	2	1	1
6	The help that my relative needs rests too heavily on my shoulders	1	−2**	0**	1
7	I became closer to my relative during the period that I was providing care	0**	3**	1**	−1**
8	Healthcare professionals take sufficient time to answer my questions	−1	2**	−1	0*
9	My relative’s situation is a constant preoccupation	*2*	*3*	*3*	*3*
10	I find it difficult to acknowledge that I also need support or help	*0*	*1*	*1*	*2*
11	I need more support in maintaining my social contacts	0	−1	−2	−2
12	I need more support with getting a break from caring overnight	0*	1*	0*	−2**
13	I need more support with equipment to help care for my relative	−2	0	−1	−2
14	I can do it myself, I don’t need support from healthcare professionals or volunteers	−1	1**	−3**	−1
15	There is room and attention for my relative’s habits and my own habits	−1	2**	−2	0**
16	It would be good if you could talk to home-care staff and/or volunteers separately, not in the presence of the sick relative	1	0	1	1
17	The arrangements for the support of family caregivers fit my needs well	−2**	1*	0	0
18	My independence is suffering	0	0	−1*	1
19	My involvement with my relative means that I feel very tied	1	1	1	2
20	Healthcare professionals listen to my relative’s wishes and my own wishes	0**	2**	−2**	1**
21	Combining the responsibility for my relative and for my job and/or family is not easy	*1*	*1*	*0*	*0*
22	I need more support with managing my relative’s symptoms, including giving medicines	−3**	0	3**	0
23	I always have to be available for my relative	2	−2**	2	3**
24	I need more support with understanding my relative’s illness	−3	−2	2**	0**
25	I want more support in dealing with (family) conflicts	−1	0**	−1	− 1
26	I need more support with providing personal care for my relative (e.g. dressing, washing, toileting)	−1	− 1	0*	−3**
27	I want to be more involved in decisions regarding the care for my relative	0	−3*	−1	− 1
28	I need more support with talking with my relative about his or her illness	−2	− 2	2	1
29	I would like to be better prepared for what is going to happen in the future, so I will know how to react to situations	0	−1	2**	− 1
30	I need more support with having time off	1**	−1	−2	2**
31	Generally speaking I felt very pressured because of the situation of my relative	1	0**	1	2
32	I need more support with dealing with my feelings and worries	1	−3**	0	1
33	It would help me to get in touch with people in a similar situation through a platform	−1	−1	0	0
34	I need more support with looking after my own health	0	−1	−1	0
35	I would like an assigned contact person who I can ask for advice if necessary	2	−1	1	0
36	I want more coordination between family members, friends or acquaintances, so I can better share the care tasks with others	−2	0	−1	− 1
37	I think it is important to get appreciation and recognition for my role and know-how	2**	0	0	0
38	I want more information about what will happen after my relative dies	*−1*	*0*	*−1*	*−2*
39	I need more support with my beliefs or spiritual concerns	−2	− 2	−3**	−1
40	I appreciate it if someone asks me if I can carry on caring for him/her like this	1	1	0	1
Explained variance (%)		10	11	10	9
Eigenvalue		4.10	4.51	4.10	3.69

Note: Profile 1 = Wants appreciation and contact person, 2 = Wishes for supportive relationships, 3 = Wants guidance, information and practical/medical support, 4 = Needs more time off. Distinguishing statements are marked as follows: *P < 0.05, **P < 0.01. A factor array of “+ 3” indicates that a family caregiver in that profile would most agree with that statement and a factor array of “-3” score that he or she would most disagree with that statement. The statements with factor arrays in italics did not distinguish between any pair of factors (these are the consensus items)

**Table 4:** The main characteristics of the four profiles

**Component**	**Profile 1** Appreciation and contact person	**Profile 2+** Supportive relationships	**Profile 2-** Omission of supportive relationships	**Profile 3** Guidance, information and practical/medical support	**Profile 4** More time off
Support needs to enable caregiver to provide care (co-worker)	Assigned contact person to coordinate care	Sharing care and shared decision-making	Sharing care and shared decision-making	Information and guidance (care coordination and illness trajectory) and practical/medical support (managing symptoms and medication)	Minimal; refrains from asking for help
Support needs for caregivers themselves (co-client)	Advice and listening ear	Take over care at night	Support with own feelings and worries, and take over care at night	No support needs expressed	More time off
Experienced support	Feels undervalued and wants more appreciation	Positive. Receives sufficient support from multiple sources	Negative. Feels neglected by healthcare professionals	Critical: wishes are not fulfilled by healthcare professionals	Critical: involvement of more professionals is at the expense of own privacy and quality of care
Experiences with caregiving	Providing care feels good	Care enhances relationship with relative	Feels overwhelmed and alone	Providing care is satisfying but not always easy	Care is demanding, and struggling with changing relationship with relative
Caregiver capacity	Can cope well with care, but needs empowerment	Can cope well with care and manages care pretty well	Struggling with care on their own	Can cope with care, but needs support to continue caregiving	Can hardly cope with care and experiences a heavy burden

### Profile 1: wants appreciation and a contact person

Taking care of their relative or friend gives family caregivers fitting this profile a good feeling [1], but they find it difficult to set boundaries and stand up for themselves [2] *“It’s difficult because I was so busy with my parents and thinking on their behalf that well, it never stopped. Because they weren’t able to do things any more. I became part of them, I didn’t feel I was myself any more: no, I’d become an extension of my parents.”.* They think that the available arrangements for support do not fit their needs well [17] *“There’s no support at all. No. You have to do it yourself as a family caregiver. I don’t feel I get any support as a family caregiver. You get shunted from pillar to post for everything.”.* They would like to have an assigned contact person [35] *“I really feel the need for a permanent contact person. I also always tell them my entire story. So I think it’s important for me to have a permanent contact. Things go wrong when there are several people involved.”.* Also, they would like to receive more appreciation and recognition for their role and know-how as a caregiver [37]*“Definitely! We are undervalued. Sorry, but there’s very little in the way of recognition.”.* These family caregivers seek a listening ear, a sparring partner and advice about the care situation. However, they do not want more medical or disease-related support, such as support in managing symptoms [22] *“I certainly didn’t need any support in dealing with medicines, for example, because I already knew all about that.”.* Nor do they want help in understanding their relatives’ illness [24].

Nine family caregivers fitted this profile. They provided care for partners, parents and other relatives with a variety of diseases (dementia, cancer, COPD, heart failure and ALS). It is notable that the family caregivers in this profile often worked in the care sector.

### Profile 2: supportive relationships

This profile is bipolar because it is defined by both positively and negatively loading Q-sorts. This means that the profile consists of opposing support needs (a family caregiver whose Q-sort is positively associated with this profile would rank a certain statement as one they ‘most agreed’ with, whereas a family caregiver whose Q-sort is negatively associated with the profile would rank this statement as one they ‘most disagreed’ with [29, 31, 32, 40]. However, the central need in both profile poles is supportive relationships. Family caregivers in the positive pole have supportive relationships, including the relationship with the care recipient, support from other family caregivers, and support from healthcare professionals. For family caregivers in the negative pole this is a wish that is not fulfilled. Hence, this profile consists of two opposing poles who have the same needs but different experiences. This profile can therefore be described in two ways [32].

#### 2+ supportive relationships

Family caregivers in the positive pole of this profile can often share the care tasks with other family members and do not feel that the help their relative needs rests too much on their shoulders [6] “*No. Like one of my brothers who said, ‘You don’t need to do all the doctor’s visits. Just say when and I’ll see if I can get time off. And another brother who lives further away and once spent the night here. It was fantastic how that went; it really happened quite automatically. Whereas we didn’t have that close a relationship, but mum was our mainstay, it was really fantastic. Everyone loved her.”.* They do not feel like they always have to be available for their relative [23]. These family caregivers also experience intimacy and positive aspects of the care situation; they become closer to their relative because of the care [7] *“The further his dementia progressed, the more he liked to be cuddled. He wasn’t really the cuddling type. And now when we sat next to one another and I put my arms around him, he’d sit there really enjoying it. […] Well, we’d never cuddled as much as this before. Yes, really special.”.* In general, they are satisfied with the contact with healthcare professionals. Healthcare professionals generally take sufficient time to answer questions [8] and listen to their wishes [20]. They do not wish to be more involved in decisions about their relatives’ care [27] *“They really got me involved. I needed that but it happened too.”.* Also, they find that there is room and attention for their relative’s habits [15] and they think there are sufficient arrangements for their support [17]. These family caregivers do not need more support in dealing with feelings and worries [32].

#### 2- omission of supportive relationships

Family caregivers in the negative pole often provide care alone, and the help their relative needs rests too much on their shoulders [6] *“The fact that I was having to do it all alone is definitely the most important aspect. Because if I’d had more time, I might have been able to look for more help. Perhaps we could have done more for him then.”.* They are not satisfied with the healthcare professionals as they do not take enough time to answer questions [8] *“They [mental health services] did come to my house but they didn’t have enough time to answer my questions. Then someone else would call from a different team and they didn’t know what had been agreed. […] It was really uncoordinated and they just blamed it on one another.”.* Healthcare professionals do not listen sufficiently to their wishes [20] and pay little attention to their habits [15], while they would welcome being more involved in care-related decisions [27] *“Yes, with the general practitioner too – that they should have got me involved earlier rather than in the final stage and then you get all that to deal with. And I just find that… well it went totally wrong and I just find that a pity.”.* These family caregivers need more support in dealing with their own feelings and worries [32] and would like to have someone take over the care at night [12].

This profile was defined by six family caregivers who provided care for partners, parents and other relatives with a variety of diseases (cancer, dementia, heart failure, COPD and stroke). Of the six family caregivers, two loaded on the negative pole of the profile, and four loaded on the positive pole. It is notable that those in the positive profile pole all received help from their own network or home-care staff, while those in the negative profile pole did not receive professional support or received no help at all.

### Profile 3: wants guidance, information and practical/medical support

Family caregivers fitting this profile need support from healthcare professionals or volunteers and can’t manage on their own [14] “*I did actually need help. I just think: you can’t do everything on your own. […] So if you have a relative with a severe illness, you won’t manage on your own. You’ll always need other people, even if it’s only to let off steam. Or so that you can say this is what I’ve run into, I’ve got this problem and what can I do about it? How should I deal with it?”.* However, these family caregivers are not satisfied with the current support as healthcare professionals do not listen sufficiently to their wishes [20] *“Right, I had to sort out the things I wanted myself. So I saw, she needs an over-bed lifting pole because that’ll let her get out of bed independently. Well, I did an awful lot… but they [healthcare professionals] didn’t help me find solutions. I did point this out, but well, they didn’t give much of a response, shall I say.”.* Family caregivers in this profile need disease-related support in managing symptoms [22] and understanding their relative’s illness [24] *“Of course when there’s a CVA, they can’t say at all what’s up but we also didn’t get nearly enough explanation about that. Not from the hospital and not from the doctors either. […] I couldn’t understand my husband at all any more. Not only was he paralysed on one side, he also reacted completely different, which I found incredibly difficult. Then you get irritable with one another, whereas if there’d been more of an explanation, if I’d known more about it, it would have been easier for me to deal with.”.* They also seek more security in the care situation: they want to be better prepared for what they could expect in the future, so they could respond better to situations [29] *“They did always say, ‘We can’t predict what will happen’. After his operation we got so much trouble from the side-effects from the medication and we couldn’t sleep, and this wasn’t really communicated properly. You have to discover everything yourself first.”.* This concerns the practical coordination of care as well as the illness trajectory and associated symptoms. Family caregivers in this profile mainly need more guidance, information and practical/medical support. They do not want support with beliefs or spiritual concerns [39].

Eight family caregivers comprised this profile. They cared for partners, parents and a friend, with a variety of diseases such as cancer, stroke, COPD, heart failure, dementia and overall decline.

### Profile 4: needs more time off

Taking care of their relative does not give these family caregivers a good feeling at all [1] *“Well, it didn’t give me a good feeling at all. Sure, it would have given me a good feeling if she loved me. But she didn’t love me anymore, she wasn’t there for me. Despite the fact that I’d been married to her for 55 years… Well, I said I’m going to finish the job but it wasn’t like I was saying great, I think it’s fantastic caring for her. It might sound hard-hearted but of course that’s how it was.”.* They feel pressured by their relative’s situation [31] *“It doesn’t feel good anymore, the relationship is so skewed now. The balance has changed completely. […] It’s really tough. It’s an awful life.”.* They also always have to be available for their relative [23] *“Right, I always had to be on call. She didn’t rely on anyone else, she only relied on me. I found that really difficult sometimes. There’ve been times when I walked away in tears: I found it that difficult. Because you always have to be there and she didn’t want help from anyone else. That was the most difficult thing for me, that she didn’t want help from anyone else. The only person she relied on was me.”.* They feel tied by their involvement with their relative’s care [19] and would like to have more time off [30] *“That involvement means that you get… you feel more tied. And that your independence starts to suffer and you always have to be on call for the person you’re caring for. That is too much. That’s the workload that gradually increases so then you start feeling the need for more free time.”.* Yet these family caregivers do not want more support with providing personal care [26] *“No, because I’d like to be alone more. Because that’s a huge infringement of your privacy as well. I mean just the fact that they come for the shower, I’m really pleased about that, but one time they come at 8:30, the next time at 9:00 and then at 10:00. So I’m always thinking: can I take a shower before they come or do I have to wait until afterwards? That too… oh no, they’re there already, OK, I’ll just wipe my face with a flannel and I’ll shower afterwards. But you do pay a very high price yourself as a result.”.* The interviews revealed various reasons for their reluctance to involve more professionals: they believe that involving more professionals is at the expense of their privacy, that their relative would not accept help from others, or they are not satisfied with the current support and therefore do not want more help. Also, these family caregivers do not want others to take over the care at night [12] *“No, see: first of all, I don’t have the room here to have someone in my home. Because if I had, I’d have started sleeping in the guest bedroom myself ages ago. And at night, well he gets out of bed once, twice or sometimes three times, but I’m now so used to it that I notice him getting up and I subconsciously know that he’s come back again, and if it takes too long, I go and take a look. So I really don’t need that.”.*

Four family caregivers were associated with this profile. They provided care for their partner with COPD, dementia or a stroke.

### Differences and similarities between the profiles

Although family caregivers in all the profiles expressed support needs, the preferred type of support differed (see Table 4). In profile 1, the main support need was an assigned contact person with whom they could coordinate care. Family caregivers in profile 2 wished for supportive relationships and valued shared decision-making with professionals. Family caregivers who wanted more guidance, information and practical/medical support (profile 3) had the most co-worker support needs, as they felt that they needed more support from healthcare professionals and volunteers [14], wanted more support with managing symptoms and giving medication [22], knowing what is going to happen in the future [29], understanding their relative’s illness [24] or talking about the illness [28]. In this respect, family caregivers in profile 3 differed most from those in profile 4 (who had no prominent co-worker support needs) and those profile 1 (who did not want practical/medical support at all).

Respite care was the most important co-client support need in profile 2 and profile 4. However, the two profiles wanted different types of respite care. Family caregivers in profile 2 (both the positive and the negative poles) would like to receive support with care overnight [12], while those in profile 4 wanted more time off [30] but not at night. These profiles differed most from family caregivers in profile 3, who did not have prominent co-client support needs. Further, family caregivers in profile 1 and profile 4 wanted some support in dealing with feelings and worries [32], while those in profile 2+ did not.

Family caregivers in the four profiles also had different experiences with caregiving. Those in profile 2+ seemed to cope with the care situation pretty well, as they did not feel burdened by the care situation [6, 23], had become closer to their relative [7] and were satisfied with the arrangements for the support of family caregivers [17] and contact with healthcare professionals [8, 15, 20]. Family caregivers in profile 1 and profile 3 also seemed to be managing the care pretty well, but those in profile 1 did not think that the arrangements for supporting them fitted well with their needs [17] and those in profile 3 were not satisfied with the contact with healthcare professionals [15, 20]. In contrast, family caregivers in profile 4 had a hard time coping with the care they had to provide. They experienced the greatest burden [23, 31], providing care did not give them a good feeling [1] and they had not become closer to their relative [7].

There were also some similarities between the profiles. In all four profiles, the relative’s situation was a constant preoccupation [9]. Furthermore, in all profiles, there were no pronounced opinions about the statements regarding difficulty in acknowledging support needs [10], the combination of responsibility for their relative and their job and/or family [21] and information needs about what will happen after their relative dies [38].

## Discussion

This study identified four distinct profiles of family caregivers of patients at home at the end of life: those who want appreciation and a contact person (profile 1), those who need supportive relationships (profile 2+ who have supportive relationships and profile 2- who lack supportive relationships), those who want guidance, information and practical/medical support (profile 3) and those who need time off (profile 4). The profiles identified family caregivers who shared similar support needs and experiences with caregiving as well as differences between the profiles. Overall, they showed similar characteristics to previously identified caregiver profiles, in that they could be roughly subdivided into family caregivers who can cope well with the care tasks [22], enjoy sufficient support and manage pretty well [17] (profile 2+), family caregivers who can cope with the care but need support in order to carry on with caregiving [22] (profile 1 and 3), family caregivers who experience care as demanding and receive little support [22] (profile 2-), and family caregivers who experience the care as a burden and can hardly cope with the care that is required [22] but do not ask for respite care [17] (profile 4).

Consistent with prior findings, acknowledgement of the family caregivers’ role and expertise would improve the situation for caregivers in profile 1 [8, 45]. They felt undervalued, and more understanding and recognition by healthcare professionals and their social environment could function as support [22]. Also, they seemed to have communication or coordination problems with healthcare professionals. Care communication and coordination appeared to be an issue for family caregivers in other profiles as well. Those who did not have supportive relationships (profile 2-) and those who wanted more guidance, information and practical/medical support (profile 3) were generally dissatisfied with the contact with healthcare professionals. However, they were dissatisfied for different reasons. As found previously, those in profile 3 felt that professionals did not have sufficient time to answer their questions and they wanted more information about managing symptoms, the disease prognosis and being prepared for the future [45]. Family caregivers in profile 2- were dissatisfied because there were hardly any professionals involved in the care and when they were involved, communication about decision-making and the coordination of care were not satisfactory. Conversely, those who had supportive relationships (profile 2+) had few unmet support needs and were satisfied with the cooperation with professionals. In line with prior work, those who were more satisfied with their role in decision-making and communication with healthcare professionals had more positive experiences with the care situation and felt less pressured (profile 2+). This underlines the importance of good communication and coordination between healthcare professionals and family caregivers [8].

In other studies, family caregivers who needed more time off (profile 4) were at risk of a heavy burden, particularly when they had no alternative solutions for the provision of care [8]. The discrepancy between the lack of support opportunities and the desire for more free time might increase the burden for these family caregivers. Hence, discussing respite options could be a way of alleviating the care-related burden [17]. It is notable that all family caregivers in this profile were caring for their partner. Some noted that they struggled with the imbalance of their relationship as it shifted from an equal husband-wife partnership to a caregiver-patient relationship. Comparable feelings were previously found among dementia caregivers [22], but can thus also occur in family caregivers caring for patients with other illnesses.

Overall, this study confirms the heterogeneity among family caregivers of patients at the end of life, as each profile group was dealing with different challenges and had different support needs [9]. Even within the profiles, no two care situations were exactly the same. Thus, caregiver-targeted support is required, while at the same time individual differences and preferences should be kept in mind.

### Methodological considerations and implications for research

In interpreting the profiles, we have to bear in mind that the family caregivers were recruited via the channels of national organizations for family caregivers and volunteers in palliative care. Hence, the majority of family caregivers who participated in this study had already found their way to some support. However, the consequences might be limited as some family caregivers reported they did not receive formal support, and some received no support at all. Concomitantly, there might be family caregivers who were under too much stress to participate, so the degree of burden may be underestimated. Also, some family caregivers may have been missed as they thought they did not meet the selection criteria because they did not recognize themselves as caregivers or were not aware of the fact that their relative was in the last phase of life [46]. Hence it is possible that we did not capture all existing views on support needs. However, we obtained a diverse sample of family caregivers, male and female, from different age groups, cultural backgrounds and work situations, with variation in illness type, relationship and contact frequency.

At the end of the interview, family caregivers were asked whether there were any additional support needs not reflected in the Q-sample. Most caregivers were satisfied with the statements in the Q-sample. One caregiver named support with feelings of guilt and another caregiver named support in accepting that the care recipient was going to die. Although these specific support needs are to some extent covered in item 32 (support with dealing with feelings and worries) it is possible that not all support needs were adequately captured in the Q-sample.

This study included both current and recent family caregivers. It could be that the recent caregivers reflected differently on needs and support received than the current family caregivers, as they assessed their needs in retrospect with possible interference of grief. However, we did not find noticeable differences between recent and current caregivers in their ranking of the statements and all profiles comprised both recent and current caregivers, thus the differences might be limited.

More than half of the family caregivers were employed at the start of their caregiving. A considerable number of them adjusted some aspect of their work situation because of the care demands. Still, not everyone was able to reconcile work and care demands, and some eventually quit their jobs. Given the ageing society and the emphasis on increasing labour market participation and raising the retirement age, in the near future even more people will have to combine work with family care [47]. This raises the question of how these working family caregivers can be supported in such a way that they can remain active in the labour market. Future research could assess the relationship between support needs and the ability to combine work and care for patients at the end of life. More knowledge about this could help employers and healthcare professionals to give working family caregivers better support.

### Practical implications

Each profile could have several implications for practice. Healthcare professionals and volunteers who deal with family caregivers who want more appreciation and a contact person (profile 1) could show that they value their role and knowledge, and involve them as a key player in the care team. It is also important to discuss who the assigned contact person should be. In addition, healthcare professionals could offer a listening ear, consider alternative support options (such as courses, peer support or support groups) and offer guidance in setting boundaries.

Family caregivers who had supportive relationships (profile 2+) received sufficient support from their social network and professionals. However, there was some need for taking over care at night. Hence, healthcare professionals and volunteers could assess whether these caregivers receive proper support both during the day and overnight. In contrast, family caregivers who did not have supportive relationships (profile 2-) felt pressured and wished to receive more support from others, and be more involved in shared decision-making and communication with professionals. Notably, none of these caregivers received formal support. It is possible that healthcare professionals are overlooking these family caregivers as they are ‘off the radar’ for home-care staff. Hence, it is important that other professionals such as general practitioners also pay attention to family caregivers of patients at the end of life and assess whether they are receiving sufficient support.

Family caregivers who wanted more guidance, information and practical/medical support (profile 3) were not satisfied with the healthcare professionals involved in their case. Healthcare professionals and volunteers should therefore be particularly alert to what these family caregivers need regarding practical and medical care for their relative. Additionally, they could provide more information about the illness trajectory and managing symptoms or giving medication. Also, it is important that they are aware of support requirements in other domains, such as talking with the family caregiver and relative about the illness and what they can expect in the future. They could take more time to answer questions and try to accommodate the wishes of these family caregivers and their relatives.

Family caregivers who wanted more time off (profile 4) found the care situation a burden. Healthcare professionals could discuss respite options (e.g. day care or involving volunteers) with these family caregivers. In addition, tips for ‘marking’ leisure time or tips for relaxing could be useful. Because family caregivers in this profile found it rather difficult to ask for help, healthcare professionals could explore the possibilities for involving members of their social network who could help with caregiving.

Although the profiles provide a good picture of the array of experiences and support needs of family caregivers who care for patients at the end of life, this does not mean that family caregivers always completely identify with only one profile. It is possible that someone recognizes something of himself or herself in multiple profiles. In addition, about one-third of the Q-sorts of the family caregivers did not load significantly on any of the four distinctive profiles. We have examined the interviews of these participants on additional views, however, overall, their explanation for the ranking of the statements was not very strong or extensive. In addition, from a theoretical perspective, there is no reason to assume that these participants would have very deviating support needs or experiences compared to the participants in the four profiles. Factors within Q-methodology are only constructed by strong and distinctive opinions. For this reason, the profiles should not be used to ‘label’ individual caregivers. They should be used primarily to make healthcare professionals and volunteers aware that family caregivers vary in their support needs and experiences with caregiving. Hence, the profiles could be incorporated in training programs for healthcare professionals or volunteers to provide an example of profound support needs of family caregivers for palliative patients, and train them how to assess and respond to these needs. Also, the profiles should be considered dynamic, as support needs and experiences may change over time and could be related to the course of the illness trajectory or other situation-specific factors. Thus, it is important to repeatedly assess caregivers’ needs. Education and training in the assessment of caregivers’ support needs could therefore be helpful, considering that it might sometimes be difficult for healthcare professionals to offer proactive support as the current allocation of help mostly depends on whether family caregivers clearly express their needs [8].

## Conclusion

To conclude, this study demonstrates that family caregivers of patients at home at the end of life have varying support needs and one size does not fit all. The results indicate that support needs sometimes remain unmet [8, 14, 46]. The profiles are relevant for healthcare professionals and volunteers in palliative care as they provide an overview of the main characteristics and support needs among family caregivers of patients near the end of life. The profiles could be kept in mind when starting a dialogue with family caregivers. This knowledge can help healthcare professionals and volunteers to provide more targeted support.

## Supplementary information

**Additional file 1.** Interview guide. This interview guide was used in and developed for the current study. It reflects the procedure and specific questions that were asked during the face-to-face interviews.

## Data Availability

The dataset used and/or analysed during the current study is available from the corresponding author on reasonable request.

## Abbreviations

ALS: Amyotrophic lateral sclerosis
CSNAT: Carer Support Needs Assessment Tool
COPD: Chronic obstructive pulmonary disease
CVA: Cerebrovascular accident
M: Mean
SD: Standard deviation
