# Supplementary material for: Profiles of family caregivers of patients at the end of life at home: a Q-methodological study into family caregiver’ support needs
Source: BMC Palliat Care. 2020 Apr 21;19:51. doi: 10.1186/s12904-020-00560-x (PMC7175554; doi:10.1186/s12904-020-00560-x)
Supplement: Supplementary file 1 — Additional file 1. Interview guide. This interview guide was used in and developed for the current study. It reflects the procedure and specific questions that were asked during the face-to-face interviews. [file 12904_2020_560_MOESM1_ESM.docx]

**Interview guide**

1. Introduction and procedure Q-study
2. **Please tell me something about the care situation?**
3. Sort the statements into three categories (agree with, disagree with, neutral)

* The statements that were used are set out in Table 3 in the manuscript

1. Allocate the ‘agreed with’ statements on the score sheet
2. Allocate the ‘disagreed with’ statements on the score sheet
3. Allocate the ‘neutral’ statements on the score sheet
4. **Are you satisfied with the current configuration of statements on the Q-sort distribution? If not, please rearrange the statements.**
5. **Could you tell me why you placed statement [number] in the ‘most agree’ column / why do you agree most with this statement?** (Ask this question for all statements in the rightmost part of the score sheet)
6. **Could you tell me why you placed statement [number] in the ‘most disagree’ column? / why did you disagree most with this statement?** (Ask this question for all the statements in the leftmost part of the score sheet)
7. **Could you tell me something about statement number .. and explain why you placed in on this spot on the score sheet?** (Ask this question for multiple statements on the score sheet. Choose these statements at random.)
8. **Are there any other statements [which are not discussed yet] that you would like to say something about?**
9. **Are there additional topics that were not included in the provided set of statements, but are also important to you as a family caregiver? If so, please explain which topics and why this is also important for you.**
